# Impact of diabetes mellitus developing after kidney transplantation on patient mortality and graft survival: a meta-analysis of adjusted data

**DOI:** 10.1186/s13098-021-00742-4

**Published:** 2021-10-30

**Authors:** Hailing Lin, Jiqiang Yan, Lei Yuan, Beibei Qi, Zhujing Zhang, Wanlu Zhang, Aihua Ma, Fuwan Ding

**Affiliations:** Department of Endocrinology, The Yancheng School of Clinical Medicine of Nanjing Medical University, Yancheng Third People’s Hospital, No. 75 Juchang Road, Yancheng, Jiangsu China

**Keywords:** Diabetes mellitus, Renal allograft, Organ transplant, Mortality, Graft failure

## Abstract

**Background:**

Post-transplant diabetes mellitus (PTDM) occurs in 10–30% of kidney transplant recipients. However, its impact on mortality and graft survival is still ambiguous. Therefore, the current study aimed to analyze if PTDM increases mortality and graft failure by pooling multivariable-adjusted data from individual studies.

**Methods:**

PubMed, Embase, and CENTRAL, and Google Scholar were searched for studies comparing mortality and graft failure between PTDM and non-diabetic patients. Multivariable-adjusted hazard ratios (HR) were pooled in a random-effects model.

**Results:**

Fourteen retrospective studies comparing 9872 PTDM patients with 65,327 non-diabetics were included. On pooled analysis, we noted a statistically significant increase in the risk of all-cause mortality in patients with PTDM as compared to non-diabetics (HR: 1.67 95% CI 1.43, 1.94 I^2^ = 57% p < 0.00001). The meta-analysis also indicated a statistically significant increase in the risk of graft failure in patients with PTDM as compared to non-diabetics (HR: 1.35 95% CI 1.15, 1.58 I^2^ = 78% p = 0.0002). Results were stable on sensitivity analysis. There was no evidence of publication bias on funnel plots.

**Conclusion:**

Kidney transplant patients developing PTDM have a 67% increased risk of all-cause mortality and a 35% increased risk of graft failure. Further studies are needed to determine the exact cause of increased mortality and the mechanism involved in graft failure.

**Supplementary Information:**

The online version contains supplementary material available at 10.1186/s13098-021-00742-4.

## Introduction

Diabetes mellitus (DM) is a metabolic disorder that is highly prevalent across the globe. Research indicates that the incidence of DM is on the rise and around 592 million people will be affected by the disease in 2035 [1]. Regardless of the progress in therapeutics and management of DM, diabetes-related complications continue to be a major healthcare problem [2].

DM is known to be an important risk factor for chronic kidney disease and end-stage renal disease (ESRD) [3]. Indeed, almost 20–38% of patients undergoing kidney transplantation have DM [4, 5]. However, a significant proportion of patients are known to develop DM after organ transplantation as well. Previously, described as “new-onset diabetes after transplantation” (NODAT), the condition is now called post-transplant DM (PTDM) in recognition of the fact that in some cases DM may have not been diagnosed before transplantation [6]. According to estimates, the incidence of PTDM ranges from 9.1 to 45.3% after 1 year, 10 to 30.0% after 3 years, and 10.2 and 15.1% after 5 years of transplantation [7]. While the traditional risk factors for DM also apply to PTDM, some specific risk factors about the transplant procedure can additionally increase the risk for PTDM [6]. In a recent systematic review and meta-analysis of 24 case–control studies, Xia et al. [8] have identified advanced age, body mass index, family history of diabetes, tacrolimus use, history of hypertension, polycystic kidney disease, acute rejection, hepatitis B virus infection, and hepatitis C virus infection as risk factors for PTDM.

Despite the intense research on the pathophysiology and risk factors for PTDM [8, 9], it is still unclear how this complication impact patient outcomes. Some studies have reported no impact of PTDM on patient and graft survival [10–14] while others indicate that mortality and graft failure is significantly increased with PTDM [15–18]. Furthermore, many of these studies have been conducted on small cohorts and may not have been sufficiently powered to detect such associations. Another important consideration is the impact of confounding variables. Mortality after kidney transplantation can be influenced by several factors like age, cardiovascular disease, DM, graft function, post-transplant urinary tract infection, and rejection treatment [19]. On the other hand, a recent study has demonstrated that older age, extended criteria donor, deceased donor, human leucocyte antigen (HLA) mismatch, and delayed graft function are risk factors for graft failure after kidney transplantation [20]. Comparing crude rates of survival not taking into account such confounding factors may therefore present false estimates.

To the best of our knowledge, to date, there has been no systematic effort to pool evidence on the impact of PTDM on patient outcomes. Given such lacunae in literature, we hereby aimed to analyze if PTDM increases mortality and graft failure by pooling only multivariable-adjusted data from prior studies.

## Material and methods

We followed the guidelines of the PRISMA statement (Preferred Reporting Items for Systematic Reviews and Meta-analyses) during the conduct of the study [21]. The review protocol was registered on PROSPERO with registration no CRD42021264402.

### Literature search

The search of relevant studies for the review was carried out electronically on the databases of PubMed, Embase, and CENTRAL. Gray literature was searched using Google scholar. To reduce single reviewer bias, two authors searched the databases independent of each other. All databases were searched from their inception. The date of the last search was 9th July 2021. We selected the following terms to explore for pertinent articles: “diabetes”, “hyperglycemia”, “kidney”, “renal”, “transplant”, “allograft”, “graft failure”, “survival”, “death”, and “mortality”. Several search queries in different combinations were conducted using Boolean operators “AND” and “OR”. Details of the search strategy common to all databases are presented in Additional file 1: Table S1. After the initial search, the results were deduplicated and the remaining articles were assessed by their titles and abstracts. We identified studies relevant to the review and extracted their full texts. The two reviewers independently evaluated these studies for final inclusion in the review. Any discrepancies in study selection were resolved by discussion with the third reviewer. In the end, we also reviewed the reference list of included studies for any missed references.

### Eligibility criteria

The inclusion criteria were formulated as follows: (1) All types of studies on adult patients (> 18 years) who had undergone kidney transplant (2) Studies comparing outcomes between patients developing PTDM and a control group with no history of DM either before or after kidney transplant (No-DM group) (3) Studies reporting data on mortality or graft failure (4) Studies reporting multivariable-adjusted outcomes with 95% confidence intervals (CI).

We excluded the following studies: (1) Studies comparing outcomes between pre-transplant DM patients with non-diabetic patients (2) Studies comparing outcomes between pre-transplant DM patients with PTDM patients (3) Studies not reporting any of the relevant outcomes and not conducting multivariate analysis for the outcomes (4) Studies reporting outcomes of patients with transient hyperglycemia (5) Non-English language studies, abstracts, case reports, and review articles. (5) Studies reporting duplicate data. In case there were multiple studies from the same healthcare setup or database, we included the study with the largest sample size.

### Data extraction and quality assessment

Data from each study was sourced by two authors independently. We extracted details of the first author, publication year, study type, study location, study database and duration, diagnostic criteria for PTDM, sample size, demographic details, dialysis modality before transplantation, duration of dialysis, type of donor, prescription of tacrolimus, follow-up duration, study outcomes, and factors adjusted for in the multivariate analysis. The primary outcome of interest was all-cause mortality while the secondary outcome of interest was graft failure.

The quality of included studies was analyzed using the Newcastle–Ottawa scale (NOS) [22]. It was conducted by two authors independent of each other. Any disagreements were solved by a discussion with the third reviewer. Studies were awarded points for selection of study population, comparability, and outcomes. The maximum score which can be awarded is nine.

### Statistical analysis

We conducted the meta-analysis using “Review Manager” (RevMan, version 5.3; Nordic Cochrane Centre [Cochrane Collaboration], Copenhagen, Denmark; 2014). We extracted adjusted hazard ratios (HR) for all-cause mortality and graft failure between PTDM vs no-DM groups from the included studies. These estimates were combined using inverse variance-weighted averages of logarithmic HRs in a random-effects model. Heterogeneity was assessed using the I^2^ statistic. I^2^ values of 25–50% represented low, values of 50–75% medium, and more than 75% represented substantial heterogeneity. Sub-group analysis was carried out based on criteria for diagnosis of PTDM. We visually inspected funnel plots to assess publication bias. A sensitivity analysis was carried out to assess the contribution of each study to the pooled estimate by removing one study one at a time and recalculating the pooled HR estimates for the remaining studies.

## Results

### Search and study details

**Fig. 1:**
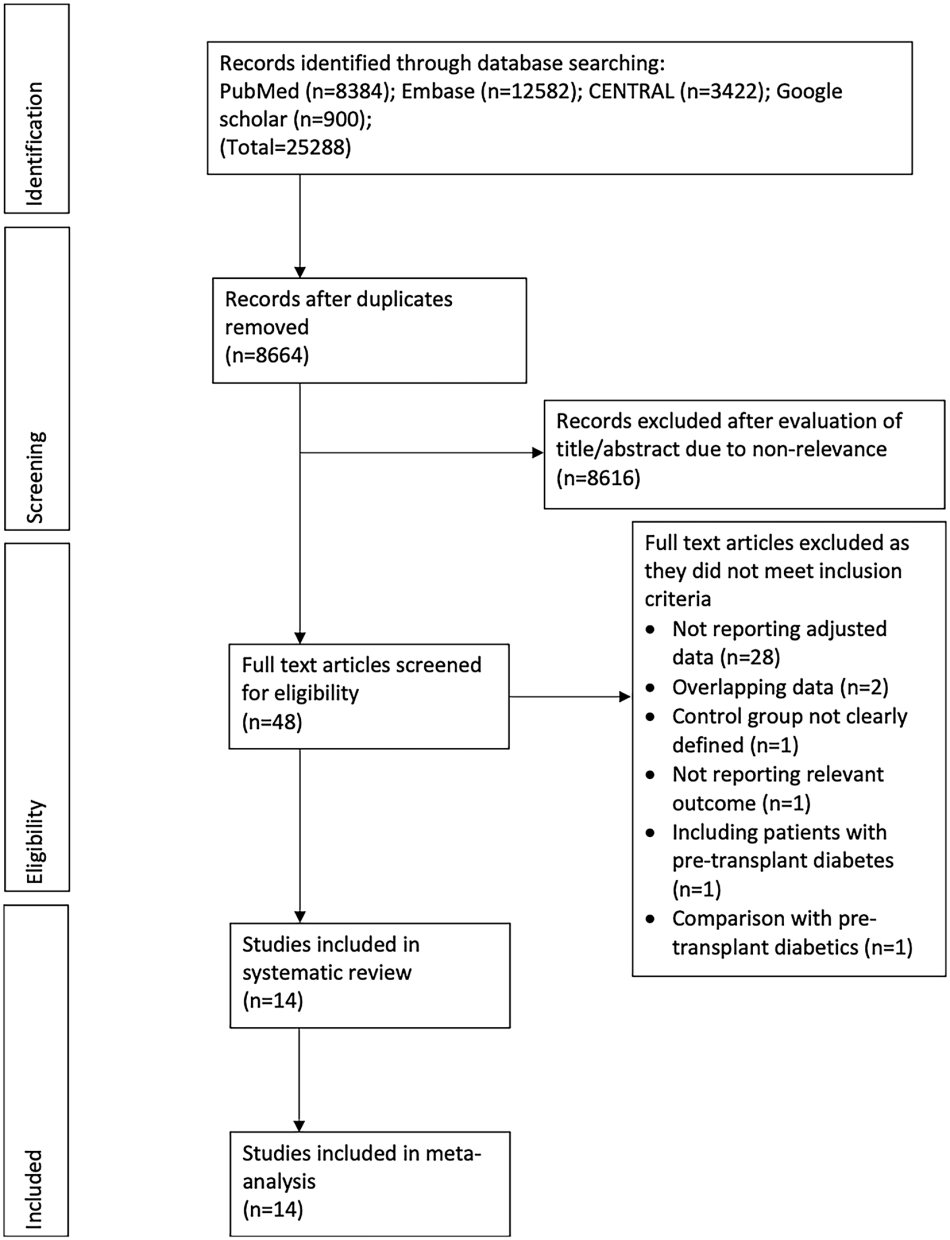
Study flow chart

The study flow chart is presented in Fig. 1. After the search and deduplication of results, a total of 8664 unique articles were assessed. These results were screened based on title and abstracts and 48 articles were selected for full-text analysis. Thirty-four studies were excluded with reasons. Finally, 14 studies were included in this systematic review and meta-analysis [15–18, 23–32].

Details of included studies are presented in Table 1. All included studies were retrospective cohort studies. Seven studies were carried in North America, four in Europe, and three in Asia. Eight of the studies used the American Diabetic Association (ADA) criteria for diagnosis of PTDM while five of them diagnosed PTDM based on the prescription of antidiabetic drugs post-transplantation. None of the studies included patients with pre-transplant DM in the PTDM group. The sample size of the PTDM group ranged from 58 to 2798 patients while that of the no-DM group ranged from 92 to 25,109 patients. In total, 9872 patients with PTDM were compared with 65,327 non-diabetic patients in the included studies. The mean age of the study participants ranged from 30.8 years to 69 years. Data on dialysis modality, dialysis duration, type of donor, and use of tacrolimus was not universally reported in the included studies. The follow-up duration was more than a year in all studies with a maximum of 10 years. On quality assessment using the NOS scale, most of the studies were high quality with a score of 8. One point was deducted from the overall score for all studies as details of the number of patients achieving final follow-up were not clear from any of the included studies. The study of Kuo et al. [32] was rated 7 because of their shorter follow-up.

**Table 1:** Details of included studies

Study	Location	Database and study duration	Diagnosis of PTDM	Group	Sample size	Mean age (years)	Male gender (%)	PD prior to transplant (%)	Dialysis duration (months)	Living donor (%)	Tarcolimus therapy (%)	Follow-up	NOS score
Yeh 2020 [17]	Taiwan	Taiwan National Health Insurance Research Database (1997–2011)	Using ICD codes	PTDM No-DM	631 2501	50.62 43.15	53.7 47.8	6.2 8.5	NR	NR	69.1 76.8	Mean 5.19 to 7.77 years	8
Aleksic 2020 [28]	USA	Montefiore Health System (2012–2014)	ADA criteria	PTDM No-DM	58 92	56 47.4	62.1 57.6	NR	NR	10.3 25	NR	Median 45.5 months	8
Dienemann 2016 [16]	USA	University of Pennsylvania (1996–2012)	ADA criteria	PTDM No-DM	447 980	51.5 46.3	60.2 56.2	NR	27.4 ± 33.9 27.8 ± 37.1	32 38.2	89.5 89.2	Mean 6.8 to 7.4 years	8
Lv 2014 [27]	China	Zhongshan Hospital (1993–2008)	ADA criteria	PTDM No-DM	87 341	45.6 38.9	69 66.9	5.7 8.8	35.3 ± 44.7 28.7 ± 36.6	9.2 27	NR	Up to 10 years	8
Wauters 2012 [15]	USA	Mayo clinic, Rochester (1984–2008)	ADA criteria	PTDM No-DM	154 992	NR	NR	NR	NR	NR	NR	Mean 90.4 months	8
Valderhaug 2011 [30] and 2012 [29]	Norway	Oslo University Hospital (1995–2006)	ADA criteria	PTDM No-DM	245 637	56 49	65 62	NR	15 ± 10 15 ± 14	33 51	13 9	Median 6 years	8
Tsai 2011 [31]	Taiwan	Chung Shan Medical University Hospital (1999–2008)	ADA criteria	PTDM No-DM	104 253	55.4 52.4	56.7 49.8	14.4 17.4	23 ± 29.2 28.8 ± 32.9	NR	82.7 76.3	Mean 70.3 to 74 months	8
Kuo 2010 [32]	USA	Organ Procurement and Trans- plant Network/United Network for Organ Sharing (OPTN/UNOS) database (2004–2007)	Using ICD codes	PTDM No-DM	2104 20,964	52.9 47.5	62.9 58.3	NR	NR	34.4 43.8	82.5 78.6	Median 548 days	7
Demirci 2010 [23]	Turkey	Ege university school of medicine (1989–2003)	ADA criteria	PTDM No-DM	102 453	36 30.8	56.9 64.3	NR	27 ± 28 20 ± 23	68.6 74.6	NR	Up to 10 years	8
Cole 2008 [18]	Canada	US renal data system (1995–2002)	Using ICD codes	PTDM No-DM	2598 25,109	NR	NR	NR	NR	NR	NR	Up to 10 years	8
Gonzalez-Posada 2006 [24]	Spain	NR (1990, 1994 and 1998)	National diabetes data group 1979 criteria	PTDM No-DM	251 2958	42.9 39.8	56.2 63	NR	34.6 ± 38 43.3 ± 46.9	NR	NR	3 years	8
Kasiske 2003 [25]	USA	US renal data system (1996–2000)	Using ICD codes	PTDM No-DM	2798 8861	NR	NR	NR	NR	NR	NR	Up to 3 years	8
Cosio 2002 [26]	USA	Ohio State University (1993–1997)	Transplanted patients who previously were not diabetic required treatment of hyperglycemia with either oral hypoglycemic agents and/or insulin	PTDM No-DM	293 1186	48 40	63 58	NR	NR	NR	NR	Up to 10 years	8

Median values

*ADA* American diabetes association, *DM* diabetes mellitus, *ICD* International Classification of Diseases, *NR* not reported, *PD* peritoneal dialysis, *PDTM* post-transplant diabetes mellitus, *NOS* Newcastle Ottawa scale

### Meta-analysis

Outcomes of interest reported by the included studies are presented in Table 2. The factors adjusted for the multivariate analysis varied widely across the 14 studies.

**Table 2:** Outcomes of included studies

Study	Outcomes assessed	Hazard ratio (95% CI)	Factors adjusted in multivariate analysis
Yeh 2020 [17]	Graft failure MACE All-cause mortality Death with functioning graft	1.75 (1.56, 1.96) 1.59 (1.38, 1.84) 1.79 (1.59, 2.01) 1.94 (1.71, 2.20)	Age, gender, Charlson comorbidity scores, place of residence, income levels, occupations, presence of comorbidities (including malignancy, hypertension, hyperlipidemia, cerebrovascular disease, myocardial infarction, congestive heart failure, peripheral vascular disease, atrial fibrillation, chronic obstructive pulmonary disease, cirrhosis, hepatitis B virus, hepatitis C virus), cyclosporine, tacrolimus, mycophenolate mofetil, mammalian target of rapamycin inhibitor, steroid, kidney transplantation rejection and cytomegalovirus infection
Aleksic 2020 [28]	All-cause mortality	1.40 (0.23, 8.51)	Age, gender, and serum creatinine one year post-transplant
Dienemann 2016 [16]	All-cause mortality Graft failure Death censored graft loss Death with functioning graft	1.57 (1.16, 2.12) 1.22 (1.00, 1.48) 1.10 (0.87, 1.39) 1.44 (1.07, 1.95)	Age, gender, ethnicity, rejection in first year, hepatitis C virus + status, delayed allograft function, living donor, glomerulonephritis, cardiovascular disease before transplant, time on dialysis, transplant year, e-glomerular filtration rate at 6 months, donor age, body mass index, cold ischemia time, HLA-DR mismatch, Panel reactive antibody status, tacrolimus as starting agent and cytomegalovirus disease
Lv 2014 [27]	All-cause mortality	1.22 (0.80, 1.84)	Age, year of transplant, postoperative onset of tumor, postoperative infection, and other risk factors
Wauters 2012 [15]	All-cause mortality	3.48 (2.25, 5.38)	NR
Valderhaug 2012 [29]	Graft failure	1.30 (0.98, 1.73)	Age, gender, body mass index, creatinine level at baseline, deceased-donor status, preemptive trans- plantation, donor age, early rejection, and early cytomegalovirus infection
Valderhaug 2011 [30]	All-cause mortality	1.54 (1.09, 2.17)	Age, gender, body mass index, creatinine, pre-transplant cardiovascular disease, total cholesterol, hypertension, and smoking status, donor status, pre-emptive transplantation, cytomegalovirus infection, early rejection, and use of cyclosporine A
Tsai 2011 [31]	All-cause mortality Graft failure	0.63 (0.13, 2.97) 0.44 (0.05, 3.73)	Age, gender, parental diabetes mellitus, hepatitis C virus, duration of renal replacement therapy, cardiovascular accident, coronary artery disease, peripheral artery occlusive disease, and hypertension
Kuo 2010 [32]	All-cause mortality Graft failure Death censored graft loss	1.22 (0.94, 1.59) 1.20 (0.99, 1.47) 1.16 (0.89, 1.50)	Age, sex, race, body mass index, primary payment, pretransplant comorbid conditions (hypertension, cardiovascular disease, peripheral vascular disease, and hepatitis C infection), duration of pretransplant dialysis therapy, serum creatinine level at 1 year, peak panel-reactive antibody titer, expanded criteria donor, HLA mismatch, induction therapy, immunosuppression at discharge, cold ischemia time (deceased donor only), and living/deceased donor
Demirci 2010 [23]	Graft failure	1.49 (1.05, 2.10)	Age, gender, acute rejection, pre-transplant dialysis duration, hepatitis C infection, cytomegalovirus infection
Cole 2008 [18]	Graft failure Death censored graft loss Death with functioning graft	1.24 (1.14, 1.35) 1.12 (0.99, 1.26) 1.41 (1.25, 1.59)	Patient demographics (age at transplantation, gender, race, cause of end stage renal disease, duration of dialysis before transplantation, body mass index, hepatitis C sero-status) and transplant characteristics (donor source, HLA mismatch, panel reactive antibody, transplant era, induction immunosuppression, and maintenance immunosuppression [medications prescribed at the time of hospital discharge after transplantation])
Gonzalez-Posada 2006 [24]	All-cause mortality Death censored graft loss	1.55 (1.05, 2.27) 1.02 (0.71, 1.48)	NR
Kasiske 2003 [25]	All-cause mortality	1.87 (1.60, 2.18)	Age, race, ethnicity, donor gender, HLA mismatch, obesity, hepatitis C, education, immunosuppression, cause of disease, transplant year, donor source (living vs. cadaver), preemptive transplantation, gender, employability, donor age, donor race, cold ischemia time, panel reactive antibody status, and other immunosuppressive agents
Cosio 2002 [26]	All-cause mortality	1.88 (1.07, 3.30)	Age, gender; serum albumin, weight, total cholesterol, triglycerides, systolic blood pressure

*NR* not reported, *HLA* Human leukocyte antigens

**Fig. 2:**
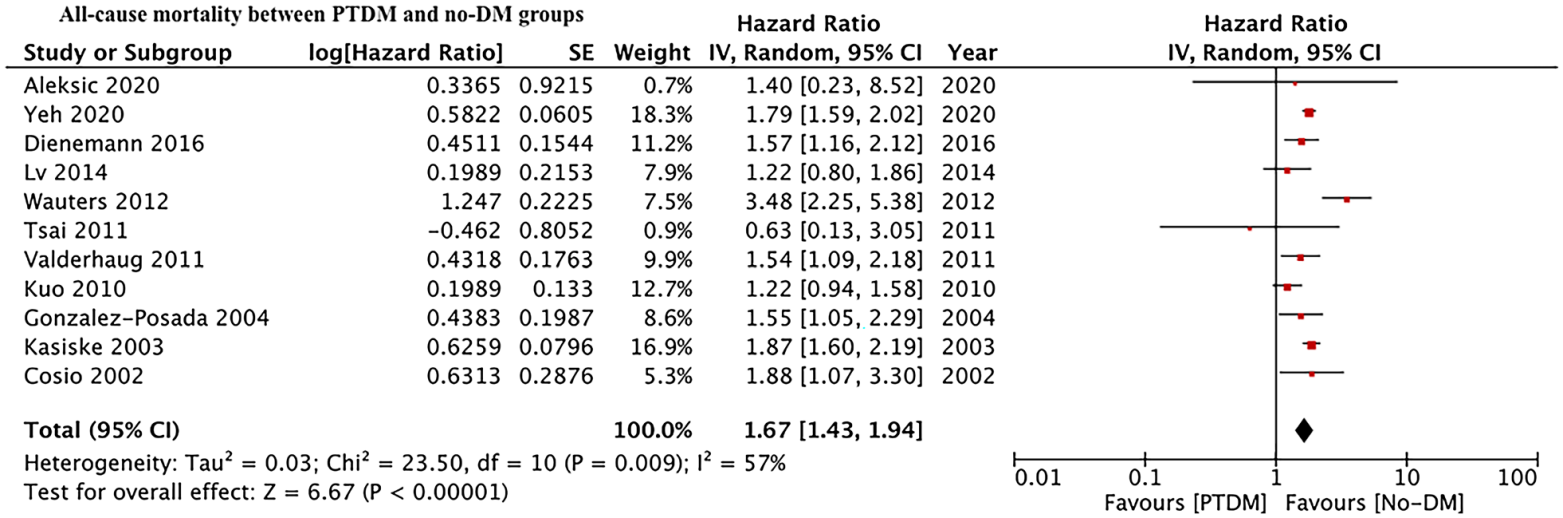
Meta-analysis of all-cause mortality between PTDM and no-DM groups

**Fig. 3:**
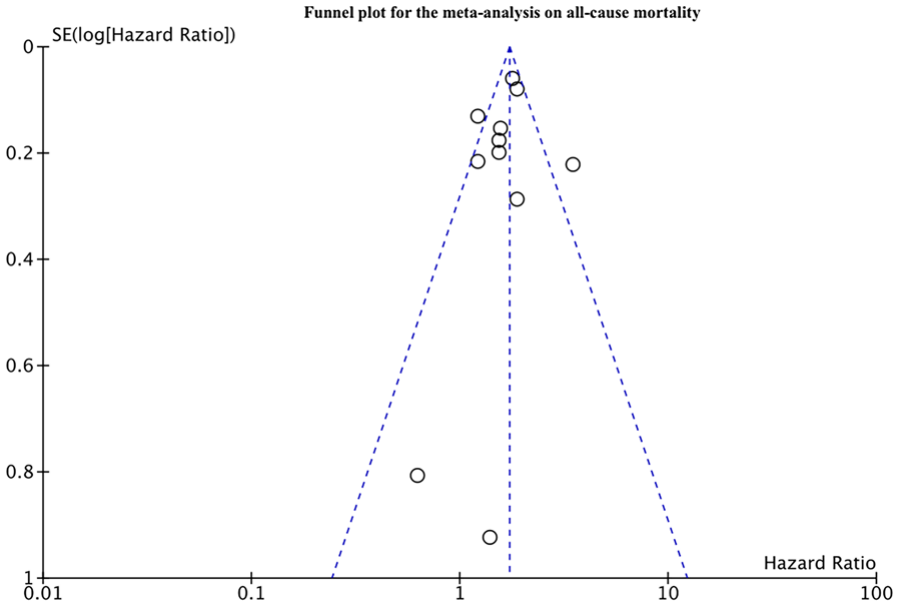
Funnel plot for the meta-analysis on all-cause mortality between PTDM and no-DM groups

Eleven studies reported adjusted data on all-cause mortality. On pooled analysis, we noted a statistically significant increase in the risk of all-cause mortality in patients with PTDM as compared to non-diabetics (HR: 1.67 95% CI 1.43, 1.94 I^2^ = 57% p < 0.00001) (Fig. 2). The results were stable on sensitivity analysis with no change in the significance of the results on the exclusion of any study. No publication bias was detected on inspection of the funnel plot (Fig. 3). Results of subgroup analysis based on the definition of PTDM as ADA criteria or non-ADA criteria are presented in Table 3. Our analysis indicates a statistically significant increased risk of mortality in PTDM patients irrespective of the criteria for diagnosis of PTDM.

**Table 3:** Subgroup analysis based on definition of PTDM

Definition	Number of studies	Hazard ratios
All-cause mortality		
ADA criteria	6	1.67 95% CI 1.16, 2.41 I^2^ = 66% p = 0.005
Non-ADA criteria	5	1.66 95% CI 1.43, 1.94 I^2^ = 53% p < 0.00001
Graft failure		
ADA criteria	4	1.28 95% CI 1.11, 1.48 I^2^ = 0% p = 0.0009
Non-ADA criteria	3	1.38 95% CI 1.08, 1.77 I^2^ = 92% p = 0.01

*ADA* American dental association, *CI* confidence intervals

**Fig. 4:**
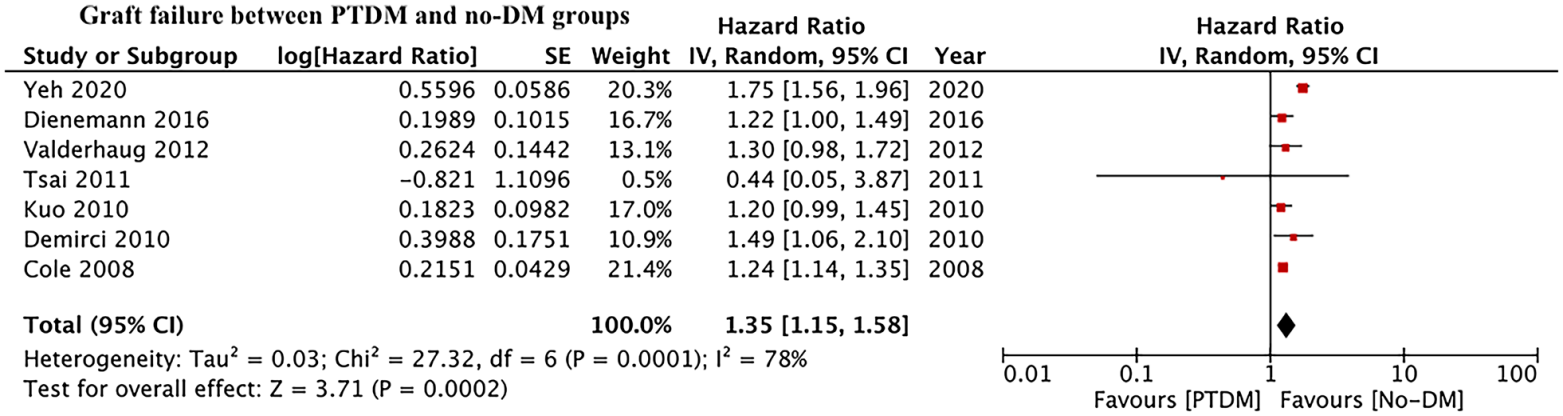
Meta-analysis of graft failure between PTDM and no-DM groups

**Fig. 5:**
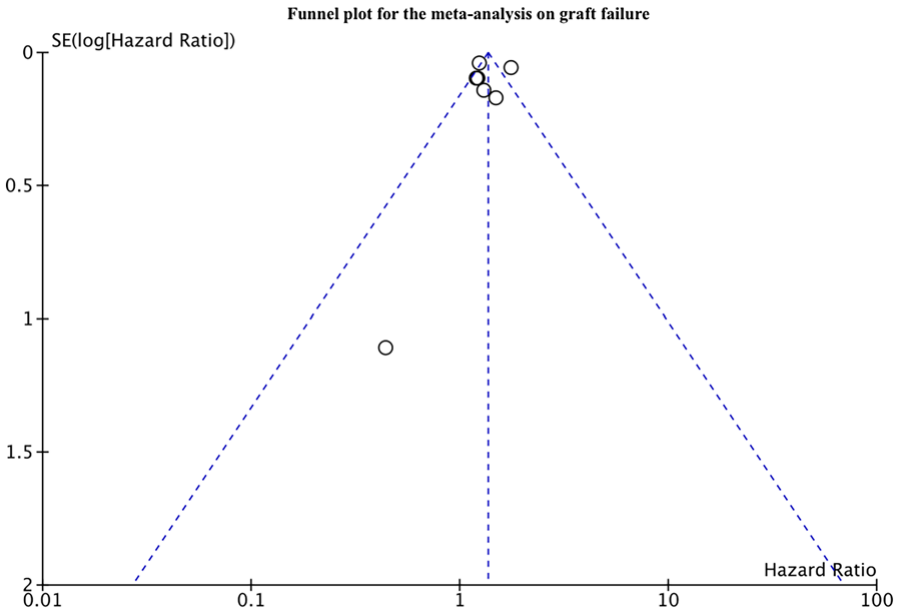
Funnel plot for the meta-analysis on graft failure between PTDM and no-DM groups

Seven studies reported data on graft failure. Meta-analysis indicated a statistically significant increase in the risk of graft failure in patients with PTDM as compared to non-diabetics (HR: 1.35 95% CI: 1.15, 1.58 I^2^ = 78% p = 0.0002) (Fig. 4). Similar results were obtained on the exclusion of one study at a time on sensitivity analysis. We noted no evidence of publication bias on the funnel plot (Fig. 5). The risk of graft failure was statistically significant for studies using ADA and non-ADA criteria for the diagnosis of PTDM (Table 3).

## Discussion

Our study is the first systematic review and meta-analysis assessing the impact of PTDM on patient outcomes. In a pooled analysis of 14 retrospective studies, we found that the development of PTDM significantly increases the risk of all-cause mortality and graft failure as compared to non-diabetic kidney transplant patients.

Over the last few decades, the role of kidney transplantation for managing ESRD has increased significantly and it can be attributed to the cost-effective nature of the treatment with lowered impact on patients’ quality of life as compared to dialysis [33]. While mortality rates of transplant patients are lower than those on maintenance dialysis, overall patient survival is still worse as compared to the general population [34]. In this context, efforts have been directed to improve patients’ survival as well as graft survival in kidney transplantation patients. An important factor impacting mortality and graft survival, which has been much discussed but never systematically reviewed, is PTDM. With conflicting results from several studies, the exact impact of PTDM on patient outcomes has never been quantified to date. In this context, the results of our meta-analysis assume clinical significance by providing true estimates of the effect of PTDM on patient outcomes. In our analysis, we noted a statistically significant 67% increased risk of all-cause mortality, ranging from 43 to 94%, in patients with PTDM as compared to non-diabetic controls. On close examination of the meta-analysis plot, it can be noted that seven of the 11 studies in the meta-analysis demonstrated a significant increase in mortality while no such difference was noted in four studies [27, 28, 31, 32]. The contrasting results of three [27, 28, 31]of these four studies may be partly explained by their small sample size. On the other hand, the lack of significant results in the study of Kuo et al. [32] may be somewhat explained by their non-standard criteria for diagnosing PTDM and shorter follow-up.

In 2003, the ADA and World Health Organization (WHO) published the first international consensus guidelines for PTDM which included the standard criteria for diagnosis of this disease. PTDM or NODAT, as per the then nomenclature, was defined as fasting glucose ≥ 126 mg/dL (7 mmol/L) on more than one occasion, random glucose ≥ 200 mg/dL (11.1 mmol/L) with symptoms, or a 2-h glucose level after a 75-g oral glucose tolerance test (OGTT) of ≥ 200 mg/dL (11.1 mmol/L) [35]. Later in the year 2014, these criteria were revised to include hemoglobin A1c as well [36]. It is quite pertinent to note that varying criteria for diagnosis can influence the outcome of PTDM. While most of the included studies used the ADA definition of PTDM some did not. However, on sub-group analysis, we still noted an increased risk of all-cause mortality with PTDM irrespective of the diagnostic criteria. Another important factor is the timing of diagnosis. Transient hyperglycemia is very common in the early period after transplantation and therefore it is recommended to delay diagnosis by at least 10 weeks [6]. While the impact of transient hyperglycemia on patient and graft survival is unclear, evidence suggests that it certainly increases the risk of PTDM in the future [37]. Few studies have reported that impaired fasting glucose without overt PTDM also increases the risk of all-cause mortality as compared to non-diabetic controls [15, 30]. This may be one of the reasons that we found a statistically significant increased risk of mortality even in studies not using the ADA definition of PTDM.

The increased risk of mortality in PTDM can be due to several reasons. Specific analysis on cause-related mortality could not be conducted in our review due to limited data. However, a few studies have analyzed cause-specific mortality in PTDM patients. Yeh et al. [17] in their study have noted a significantly increased risk of cardiovascular, infectious as well as cancer-related mortality in PTDM as compared to controls. On the other hand, Valderhaug et al. [30] have noted that PTDM increases the risk of only cardiovascular mortality but not of infection or cancer-related mortality. Because of the limited evidence, there is a need for more studies examining cause-specific mortality in PTDM patients.

Compared to all-cause mortality, the evidence on the impact of PTDM on graft failure is further unclear [9]. In our analysis, we noted that PTDM leads to a statistically significant 35% increased risk of graft failure as compared to non-diabetic controls. However, the results should be interpreted with caution as only seven studies reported data for this analysis which represent only half of the total studies in this review. On a positive note, this is the only pooled analysis of the multivariable-adjusted risk of graft failure after kidney transplantation conducted to date. On examination of the forest plot, almost all studies noted an increased risk or a tendency for increased risk of graft failure, except for Tsai et al [31]. This may be partly attributed to the small sample size of this study and more importantly to the difference in the confounding variables adjusted. It is well recognized that acute rejection and opportunistic infections especially in the first year after transplantation are important risk factors for graft failure [38]. In the study of Tsai et al [31] none of these factors were adjusted. Four of the studies in this meta-analysis included acute rejection as an adjusted factor [16, 17, 23, 29], while two studies excluded patients with acute rejection from their analysis [18, 32]. The importance of this factor is emphasized with the fact that management of acute rejection involves aggressive use of immunosuppressive drugs which in turn can lead to PTDM [16]. Cole et al. [18] in the study have separately analyzed the impact of acute rejection and PTDM on graft failure. The authors reported that while both acute rejection and PTDM reduce all-cause graft survival, the mechanisms are different, with acute rejection significantly associated with death-censored graft loss while PTDM leading to significantly increased risk of death with functioning graft and was not associated with death-censored graft loss. Similar results have been reported by other included studies in our review as well [16, 17, 24, 32]. Valderhaug et al. [29] in their study have reported no association between fasting plasma glucose or any level of hyperglycemia and graft failure. The results of these studies indicate that the negative association between PTDM and graft failure may be related to the increased mortality due to PTDM and the elevated glucose levels in PTDM may not result in irreversible kidney damage [29].

Our review has some limitations. Firstly, we understand that our analysis is not an exhaustive data analysis of available literature as a large number of studies not reporting adjusted outcomes were excluded. However, this was deemed important owing to the several confounding variables which can influence outcomes of PTDM. Secondly, an important limitation of our analysis is that the factors adjusted in included studies were not the same. The exclusion of known or unknown confounders may have influenced the study results. Thirdly, the sample size of many of the included studies was not high. The outcomes were not universally reported by all included studies and only seven studies were available for the analysis on graft failure. Fourthly, all included studies were retrospective in nature which have inherent selection bias. Studies from administrative databases or insurance records are prone to data entry errors which could skew the study outcomes. Fifthly, most of the studies were conducted on kidney transplantations done before 2010. The impact of recent immunosuppressive therapies and advances in the care and management of PTDM may have not been reflected in our results. The included studies also had variations in the definition of PTDM and the duration of follow-up. Furthermore, it is plausible to assume that the immunosuppressive protocol, the time of diagnosis, and management of PTDM would have been variable in the included studies and these factors could also have influenced outcomes. Lastly, majorities studies were from North America and this would limit the generalizability of our results.

Nevertheless, our study has some strengths. Data of 9872 patients with PTDM was analyzed in our review. The overall quality of studies on the NOS scale was high and the results were stable on sensitivity analysis which lends credibility to our results. By pooling only multivariable-adjusted data, we have hereby presented the best available evidence in the literature on the impact of PTDM on patient outcomes.

To conclude, our results indicate that kidney transplant patients developing PTDM have a 67% increased risk of mortality and a 35% increased risk of graft failure. Further studies are needed to determine the exact cause of increased mortality and the mechanism involved in graft failure.

## Supplementary Information

**Additional file 1: Table S1.** Search strategy.

## Data Availability

The datasets used and/or analyzed during the current study are available from the corresponding author on reasonable request.

## Abbreviations

PTDM: Post-transplant diabetes mellitus)
HR: Hazard ratios
DM: Diabetes mellitus
ESRD: End-stage renal disease
NODAT: New-onset diabetes after transplantation
HLA: Human leucocyte antigen
ADA: American Diabetic Association
WHO: World Health Organization
OGTT: Oral glucose tolerance test
