# Supplementary material for: Impact of diabetes mellitus developing after kidney transplantation on patient mortality and graft survival: a meta-analysis of adjusted data
Source: Diabetol Metab Syndr. 2021 Oct 30;13:126. doi: 10.1186/s13098-021-00742-4 (PMC8557540; doi:10.1186/s13098-021-00742-4)
Supplement: Supplementary file 1 — Additional file 1: Table S1. Search strategy. [file 13098_2021_742_MOESM1_ESM.docx]

Supplementary Table 1: Search strategy

| **Search number** | **Query** |
| --- | --- |
| **1** | ((((kidney[Title/Abstract]) OR (renal[Title/Abstract])) AND (transplant[Title/Abstract])) AND (diabetes[Title/Abstract])) AND (survival[Title/Abstract]) |
| **2** | ((((kidney[Title/Abstract]) OR (renal[Title/Abstract])) AND (allograft[Title/Abstract])) AND (diabetes[Title/Abstract])) AND (survival[Title/Abstract]) |
| **3** | ((((kidney[Title/Abstract]) OR (renal[Title/Abstract])) AND (transplant[Title/Abstract])) AND (diabetes[Title/Abstract])) AND (mortality[Title/Abstract]) |
| **4** | ((((kidney[Title/Abstract]) OR (renal[Title/Abstract])) AND (allograft[Title/Abstract])) AND (diabetes[Title/Abstract])) AND (mortality[Title/Abstract]) |
| **5** | ((((kidney[Title/Abstract]) OR (renal[Title/Abstract])) AND (transplant[Title/Abstract])) AND (hyperglycemia[Title/Abstract])) AND (survival[Title/Abstract]) |
| **6** | ((((kidney[Title/Abstract]) OR (renal[Title/Abstract])) AND (allograft[Title/Abstract])) AND (hyperglycemia[Title/Abstract])) AND (survival[Title/Abstract]) |
| **7** | ((((kidney[Title/Abstract]) OR (renal[Title/Abstract])) AND (transplant[Title/Abstract])) AND (hyperglycemia[Title/Abstract])) AND (mortality[Title/Abstract]) |
| **8** | ((((kidney[Title/Abstract]) OR (renal[Title/Abstract])) AND (allograft[Title/Abstract])) AND (hyperglycemia[Title/Abstract])) AND (mortality[Title/Abstract]) |
| **9** | ((Kidney transplant) AND (graft failure)) AND (diabetes) |
